# Encased Gold Nanoparticle Synthesis as a Probe for Oleuropein Self-Assembled Structure Formation

**DOI:** 10.3390/ma14010050

**Published:** 2020-12-24

**Authors:** Aila Jimenez-Ruiz, Rafael Prado-Gotor, José G. Fernández-Bolaños, Alejandro González-Benjumea, José María Carnerero

**Affiliations:** 1 Department of Physical Chemistry, University of Seville, c/Profesor García González 1, 41012 Seville, Spain; jcarnerero2@us.es; 2 Department of Organic Chemistry, University of Seville, c/Profesor García González 1, 41012 Seville, Spain; bolanos@us.es (J.G.F.-B.); agonzalez15@us.es (A.G.-B.)

**Keywords:** oleuropein, gold nanoparticles, nanosystem characterization, self-assembly

## Abstract

Stable oleuropein-coated gold nanoparticles in aqueous media were synthesized for the first time. Oleuropein (OLE) concentration in the reaction medium was found to greatly influence the outcome and stability of the resulting nanocolloid, with a marked decrease in particle size being found for the more concentrated oleuropein solutions. The protection mechanisms involved in the stabilized nanosystems were analyzed. Oleuropein self-assembled structures were found to be formed at a concentration threshold of [OLE] > 5 × 10^−5^ M, and observed through the use of CryoSEM imaging. Those structures were responsible for both the increased stability and the decrease in size observed at the more concentrated solutions.

## 1. Introduction

**Scheme 1:**
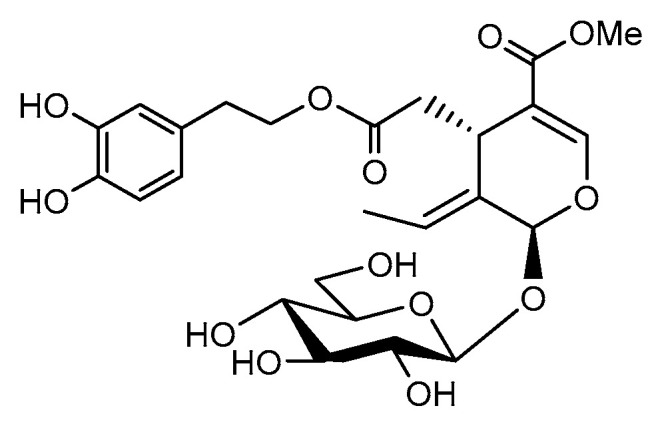
Oleuropein chemical structure.

Oleuropein, a natural phenolic compound (Scheme 1), has been found to be one of the major active components that can be isolated from olive leaves, although it is also present in olives and in the bark of the olive tree. It was described for the first time by Shasha and Leibowitz in 1959 [1], who later termed it “the bitter principle of olives” [2] due to its strong taste, which causes it to need partial or total degradation during olive processing [3]. Oleuropein is a non-toxic compound that can be isolated, among other sources, from olive processing waste [4,5], reducing the amount of residue that need to be eliminated. It has been reported to present a strong antioxidant effect [6,7,8], as well as antibacterial and antiviral properties [9,10,11], owing to its role as a natural defense mechanism. Most importantly, it possesses important anticarcinogenic properties that have contributed to placing it in the spotlight [12,13,14,15,16]. As an example, the anti-inflammatory properties of oleuropein and its potential role for the prevention and management of rheumatoid arthritis have been recently described [17].

Despite its chemical importance, the physical properties of oleuropein have received comparatively little attention. In the fields of nanotechnology and metal conjugation, although some reports exist of gold and silver [18,19] as well as non-noble metal [20,21] nanoparticle synthesis being carried out by using olive leaf extracts, no data have been reported of any colloidal synthesis using pure oleuropein as the main stabilizing agent. In the present work, we carried out a series of gold nanoparticle syntheses in the presence of varying concentrations of oleuropein. Its influence in the outcome of the procedure was analyzed, and the colloids were characterized in-depth. Results showed that some degree of oleuropein self-assembly is to be expected at the highest concentrations, and this fact dramatically affects nanoparticle morphology and stability. The analysis of those parameters for in situ synthesized nanoparticles, via simple techniques such as UV–Vis (ultraviolet-visible) absorbance, allows for them to be used as a probe for the presence of suprastructures at high oleuropein concentrations.

Gold nanoparticles (AuNPs) present interesting properties in terms of their use as colorimetric sensors, mainly due to their optical properties—the color of different dispersions depends on the size and shape of the particles. The surrounding medium (characterized by its refractive index) also plays a crucial role in the solution color [22,23,24]. In this sense, the use of UV–VIS spectra in order to monitor changes on the surface plasmon band (SPB) of non-functionalized gold nanoparticles becomes a subject of experimental interest, allowing us to obtain information about a possible red-shift of the SPB due to an aggregation process of the AuNPs [25].

## 2. Materials and Methods

### 2.1. Materials

HAuCl_4_ (Sigma-Aldrich, Darmstadt, Germany, 520918) and sodium borohydride were of analytical grade and used as received. Oleuropein was extracted using 70:30 (v/v) ethanol–water from olive leaves (*Olea europaea* L., Picual cultivar), dried at room temperature. After removing the solvent at reduced pressure, we purified the solid extract by silica gel column chromatography using a gradient elution with CH_2_Cl_2_–MeOH (10:1 → 5:1) to give oleuropein as a yellowish powder. ^1^H-NMR spectroscopic data were in agreement with those previously reported [26]. All working solutions were prepared with deionized water from a Milli-Q system, with a conductivity less than 10^−^^6^ S m^−^^1^.

### 2.2. Synthesis Procedure

Colloidal systems were obtained by direct room temperature reduction of HAuCl_4_ (at a fixed final concentration of 2.5 × 10^−^^4^ M) by sodium borohydride (2.5 × 10^−^^4^ M) in the presence of varying concentrations of oleuropein (OLE). In order to carry out the procedure, we placed 20 mL of a 5 × 10^−^^4^ M HAuCl_4_ solution in water at room temperature into a beaker, and the mixture was then magnetically stirred. Then, in rapid succession, 20 mL of OLE at double the final concentration on each case, and 0.1 mL of sodium borohydride (0.1 M) were added. Since OLE was shown to be able to start the reduction procedure by itself, the addition of sodium borohydride after reactant mixing needed to be fast in order to minimize reduction of the gold salts by OLE. Solutions turned purple to dark red, depending on OLE concentration, after mixing. The color slowly turned brown to light red in the following hours. In all cases, stirring was maintained for at least 24 h to allow for the total reduction of the gold salts. Aliquots of some of the as-prepared colloids were dialyzed on a cellulose ester dialysis membrane (MWCO = 10000) overnight.

The resulting samples were labelled as S1 to S7, and the final [OLE] in the reaction media can be found in Table 1. For control purposes, as borohydride by itself was also found to be able to stabilize the gold colloids, we obtained an additional synthesis in the absence of OLE, which was labelled as S0.

### 2.3. Absorbance Measurements

UV–VIS spectra were obtained in a Cary‑500 spectrophotometer (Agilent, Santa Clara, CA 95051 USA), with a wavelength accuracy of ± 0.3 nm and a spectral bandwidth of 0.5 nm. For sample characterization, spectra were obtained in the visible region of the spectrum, from 400 to 800 nm, and before and after dialysis, we registered spectra between 200 and 800 nm in order to monitor the UV absorption bands of the oleuropein. In all cases, temperature was kept at 298.3 K and the solvent was ultrapure Milli-Q water, with a conductivity less than 10^−^^6^ S m^−^^1^.

#### RGB Color Conversion

XYZ color space measurements were derived from transmittance values according to the CIE (International Commission on Illumination) standards for a D65 illuminant and 2º standard observer [27]. Equations for RGB color conversion from the XYZ data were obtained from https://www.easyrgb.com/en/math.php and implemented as described therein.

### 2.4. DLS and Zeta Potential Measurements

Both dynamic light scattering (DLS) measurements of colloids and oleuropein solutions and zeta potentials of the as-synthesized and dialyzed colloids were registered in a Zetasizer Nano ZS-90 (Malvern Panalytical, Spectris plc, Surrey, England) spectrophotometer equipped with a red laser (633 nm). A DTS1060 polycarbonate capillary cell was employed for all measurements. For DLS measurements, the detection was carried out at a fixed angle of 173° from the light source, and the fluctuations in scattered light intensity were analyzed. Zeta potentials were derived from the electrophoretic mobility of the sample particles. In both cases, calculations were made with the help of the Zetasizer (Malvern Panalytical, Spectris plc, Surrey, England) software.

### 2.5. TEM Measurements

Transmission electron microscopy (TEM) images of the as-synthesized colloids were obtained in a Philips CM200 (Philips Ibérica, S.A., Madrid, Spain) microscope working at 200 kV. Sample preparation was carried out by deposition of a single drop of nanoparticle suspension in a carbon-coated copper grid, which was then left to dry overnight at room temperature.

### 2.6. CryoSEM Measurements

In order to obtain cryo-scanning electron microscopy (CrioSEM) images of oleuropein solutions, we carried out sample freezing out by quick immersion in liquid nitrogen (77 K), followed by an etching process in which temperature was raised in 5 K/min steps to a final temperature of 183 K, in order to minimize ice crystal formation. A Leica EM VCT100 (Leica Camera Iberia, S.L., Madrid, Spain) contamination-free cryo transfer system was employed in order to keep the sample cooled during the whole measurement process. A 9 nm gold coating was deposited through sputtering before imaging by using a Leica ACE600 (Leica Camera Iberia, S.L., Madrid, Spain) sputter coater. Images were obtained in a Zeiss EVO L315 (Carl Zeiss Iberia, S.L, 28760 Tres Cantos, Madrid, Spain) SEM microscope at acceleration voltages of 5 kV and 8 kV.

## 3. Results

**Figure 1:**
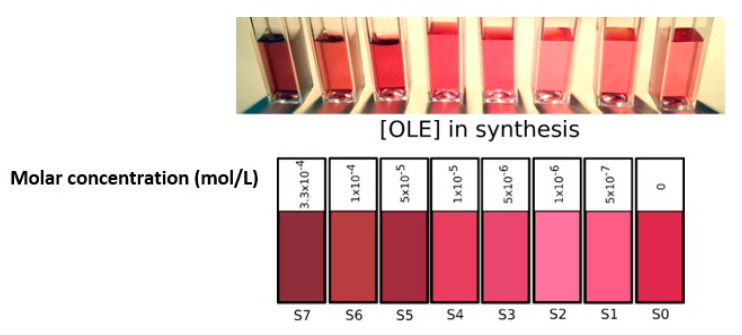
Photograph (upper) and RGB color simulation derived from absorbance spectra (lower) of all gold colloids obtained.

A photograph and a digital simulation (obtained from the conversion of their absorption spectra into RGB—see the Supplementary Materials) of the final color of all colloids resulting from the experimental procedure described is shown in Figure 1. From simple color observation from the naked eye, we could establish a clear division between the syntheses that were carried out at [OLE] > 5 × 10^−^^5^ M (S7 to S5) and those that contained lower concentrations (S4 to S1). It is important to note that, although oleuropein possessed some reducing power and was able to start gold salt reduction by itself, the resulting colloids were neither stable nor concentrated enough for analysis. The slow reduction speed observed when pure oleuropein was employed as the sole reducing agent resulted in uncontrolled particle growth (likely due to a low rate of nucleation in relation to particle growth) that caused the suspensions to turn unstable and precipitate after a short period of time. To avoid this, sodium borohydride was employed as a reducing agent. NaBH_4_ is a fast-reducing agent commonly employed for nanoparticle growth in the presence of organic capping agents, from biopolymers to proteins [28,29,30,31]. The fast reaction time linked to borohydride reactions ensured the formation of small and monodisperse particles. However, there was also a possibility of borohydride subproducts interfering with the result, as they possessed some residual stabilizing effect (as seen for synthesis S0) and could adsorb on the surface of the synthesized nanoparticles, which conferred them a negative charge and stabilized the colloid. This was an undesirable effect, and to minimize it, residual borohydride by-products should be present as minimally as possible in the final reaction product.

**Figure 2:**
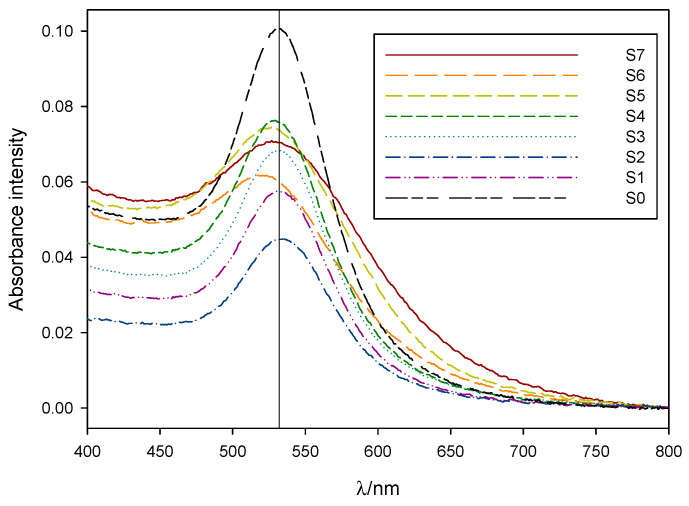
Normalized absorbance spectra, taken between 24 h and 96 h after synthesis, for 1:10 dilutions of samples S7 to S1 and S0. Vertical line marks the position for the absorption maxima of sample S0 at 531 nm.

The observed color differences between samples were clearly reflected in their respective absorbance spectra (see Figure 2). S7-5 plasmon bands were wider and slightly blue-shifted compared to that of the control sample S0, while S4-1 presented a more defined, narrower absorbance band whose peak intensity wavelength matched that of sample S0.

**Figure 3:**
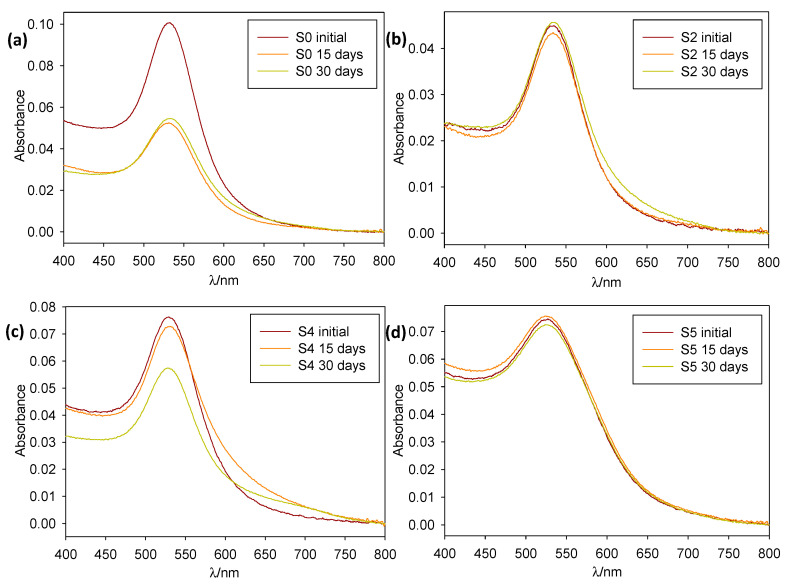
Normalized absorption spectra over time of 1:10 dilutions of samples (**a**) S0, (**b**) S2, (**c**) S4, and (**d**) S5.

The as-prepared samples were stored at room temperature and protected from light. Their stability was then monitored via absorbance spectroscopy over a four-week span time (see Figure 3). Colloid stability was found to be good, with most of them being stable (no precipitation and no notable aggregation phenomena) over a 15- or even 30-day period.

Once a nanocolloid was found to be stable for a measurable amount of time, the next step was to ascertain the exact character of the protective coating that stabilized it. Generally speaking, stabilization can be achieved through adsorption of either a charged or a neutral but voluminous ligand over the particle surface; the former procedure gives rise to positively or negatively charged nanoparticles, while the latter produces neutral colloids. A way to test for the type of stabilization of a finished colloid is to add moderate concentrations of a neutral electrolyte, such as NaCl. Electrostatically protected nanoparticles will become neutralized by the added ions and aggregate, quite often changing color in the process. Sterically protected colloids will be unaffected.

On the other hand, dialysis is one of the most common ways of eliminating undesired by-products—in this case, borohydride derivatives. This process breaks the ion exchange effect that stabilizes electrostatically protected colloids (such as commonly used citrate-capped nanoparticles) and, if they are the only source of stabilization, will result in precipitation of the colloid. Oleuropein stabilization, coming from a bulky, uncharged ligand, is expected to be due to steric effects, and therefore unaffected by dialysis.

In order to ascertain both the nature of the nanoparticle-capping agent interaction and the ionic character of the colloids, we designed two experiments. First, samples S6, S5, and S0 were dialyzed in Milli-Q ultrapure water over a 72 h period; then, the stability of all samples to 0.150 M NaCl was tested.

**Figure 4:**
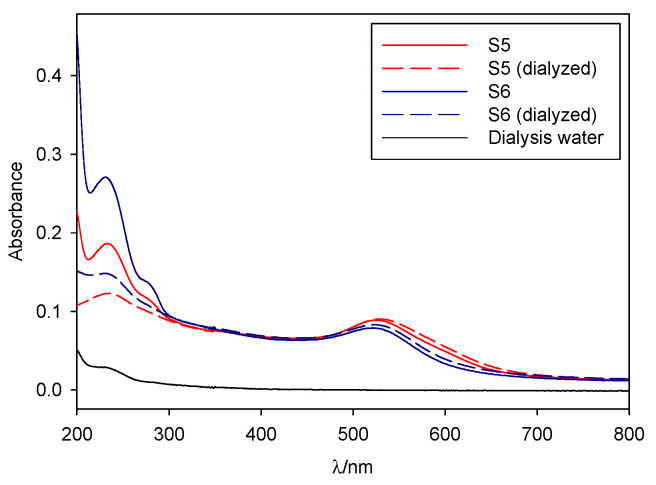
Before and after dialysis spectra for samples S5 and S6.

As seen in Figure 4, both S5 and S6 were dialyzable with a minimum loss of stability, while S0 precipitated upon dialysis. Therefore, we can infer that those two syntheses presented a markedly different character to that of pure borohydride nanoparticles, which can be attributed to the oleuropein stabilization, making them desirable for further study.

**Figure 5:**
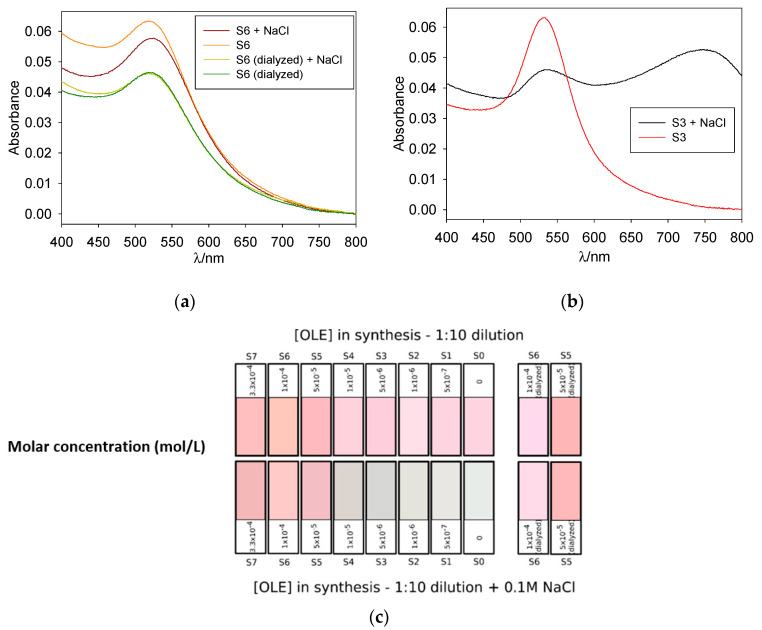
Normalized absorption spectra for 1:10 dilutions of samples S6 (**a**) and S3 (**b**) and RGB color simulation for all samples (**c**); upper row corresponds to diluted samples before NaCl addition and lower row to the same samples after an incubation period of 10 min with NaCl. Color intensity was enhanced for better perception.

NaCl acts as a good agglomerant agent for those colloids whose protection is charge-based, while having no effect over steric-protected colloids. In this way, testing for NaCl aggregation helps determine the nature of the protective coating. For gold nanoparticles, this aggregation visually manifests in the shape of a red-shifted wide absorption band (corresponding to the anisotropic multi-particle aggregates) and a shift from red to a purple or blue tint in the nanoparticle suspension; suspensions can also turn colorless when the particle size becomes big enough for precipitation. Figure 5 shows the results of the NaCl test as a RGB color simulation, and for illustrative purposes, the original absorption spectra of OLE-protected S6 (before and after dialysis) and borohydride-protected S3 are also shown. Notice the blue tint appearing for S0-S4 in the presence of NaCl, as well as the wide band over 750 nm on the S3+NaCl spectrum.

As reflected in Figure 5, samples obtained in the presence of [OLE] < 5 × 10^−^^5^ M were markedly unstable to NaCl addition, as was the blank. Almost complete precipitation of the system occurred, with the formation of an aggregation band over 750 nm and a marked color change from red to blue; the color intensity of the latter was observed to decrease with lower [OLE] as the aggregation band shifted to higher wavelengths in the infrared region of the spectrum. Undialyzed samples containing [OLE] > 5 × 10^−^^5^ M presented aggregation with a shoulder centered around 650 nm, and their color remained close to that of the original sample, although a slight darkening was observed. As expected, S5 to S7 remained stable in the presence of NaCl, with this stabilization being even clearer for dialyzed samples.

Surface zeta potential measurements were carried out for samples S6 and S5 (both dialyzed and undyalized), and S3 and S0. The results are shown in Table 2. As can be observed, no significant difference was found between surface potential of the higher [OLE] samples (S6, S5) and the one obtained in absence of oleuropein (S0), while the sample containing the lower [OLE] (S3) showed a shift towards a more neutral mean value, although it remained in the negative zone. Despite a clear diminution of the salt concentration upon dialysis, the procedure did not significantly alter the surface potential of the sample. This was a notable finding, highlighting the fact that even oleuropein-protected nanoparticles retain a slight anionic character even after dialysis. The enhanced stability to ionic aggregation of those colloids can be attributed to them not being reliant on borohydride absorption–desorption equilibria for stability, even though they are negatively charged.

### 3.1. TEM Imaging

**Figure 6:**
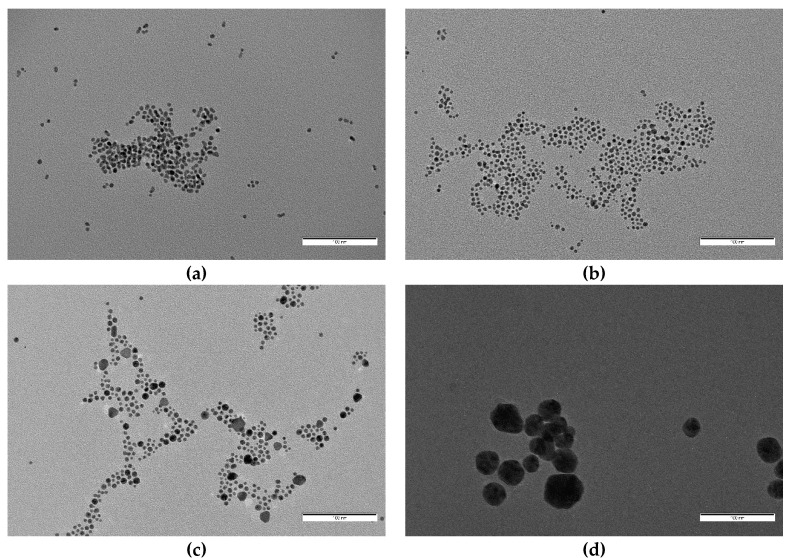

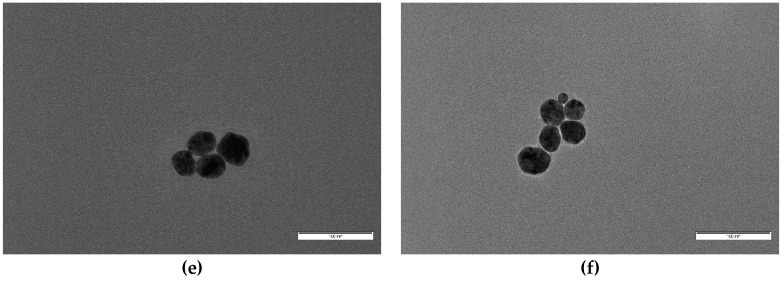
From up to down and left to right: TEM images of samples (**a**) S7, (**b**) S6, (**c**) S5, (**d**) S4, (**e**) S3, and (**f**) S0. All images were taken at the same magnification. Scale bar corresponds to 100 nm.

**Figure 7:**
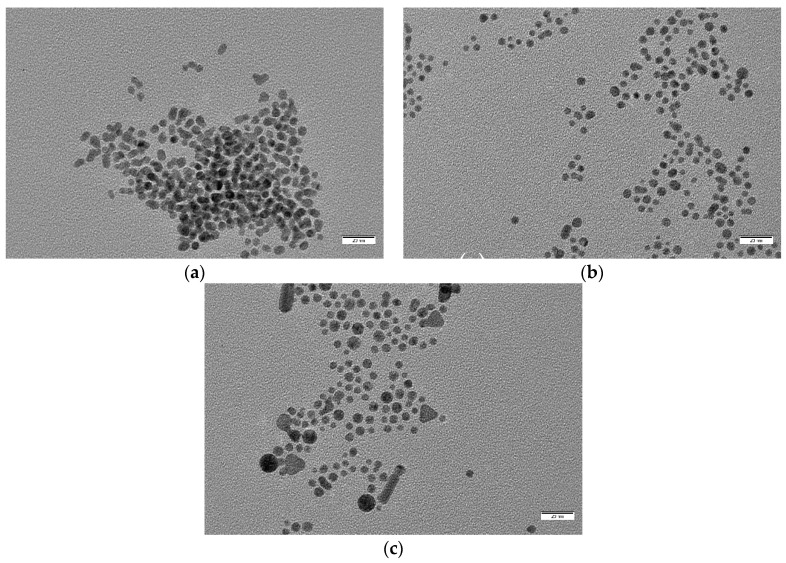
Higher magnification images of samples (**a**) S7, (**b**) S6, and (**c**) S5, showing morphology details. Scale bar corresponds to 20 nm in all cases.

For the final step of characterization, and in order to obtain accurate information about particle size and shape, we obtained TEM images for samples S7 to S3 and S0 (Figure 6 and Figure 7). TEM particle analysis by using ImageJ software also allowed us to determine mean particle size for all cases (Table 3). Again, clear differences in size and morphology can be appreciated between those samples with oleuropein concentrations over and under 5 × 10^−^^5^ M. Nanoparticles obtained in the absence of oleuropein had a mean diameter of 32.8 nm, and showed a small degree of sphericity with the presence of clear multi-faceted shapes. The same situation was observed for those particles obtained in the presence of [OLE] = 5 × 10^−^^6^ M and 10^−^^5^ M (S3 and S4), which showed a similar morphology and slightly bigger diameters (35.0 nm in both cases), the latter having a markedly greater polydispersity. S4 was also found to be the less stable of all colloids obtained (see Figure 3).

For concentrations of oleuropein of and over 5 × 10^−^^5^ M, mean particle diameter decreased dramatically and clear shape changes were observed (Figure 7). S5 nanoparticles (with [OLE] = 5 × 10^−^^5^ M) were mostly spherical, with a medium diameter of 5.6 nm, no clear faceted growth, and a small population of bigger particles (around 14 nm), alongside elongated and triangular shapes (with a mean 9.9 nm side length). S6 ([OLE] = 10^−^^4^ M) nanoparticles were slightly smaller (4.6 nm) and did not show any non-spherical shapes, although some coalesced or twinned particles were observed. Polydispersity sharply decreased from that of the more dilute oleuropein solutions. Finally, for synthesis S7 ([OLE] = 3.3 × 10^−^^4^ M), the sample presented a mean diameter and polydispersity similar to that of S6, but a sizable population of multi-twinned particles was also found alongside single spherical ones. Polydispersity was found to be around 20% for all samples, except those closer to the size transition zone, S4 (40%) and S5 (36%).

However, while S4 polydispersity can be attributed to the presence of clearly defined anisotropic shapes, S3 is due to great size variations of the mostly spherical particles that compose it. Both the decrease in particle size and the presence of anisotropic particles are common indicators of restricted nanoparticle growth phenomena, with anisotropy arising from interstitial particle growth. That the greater number of anisotropic shapes was found at the lowest OLE concentration is also notable, indicating that growth inside superestructures increases for higher oleuropein concentrations.

### 3.2. Oleuropein Macrostructure Characterization

**Figure 8:**
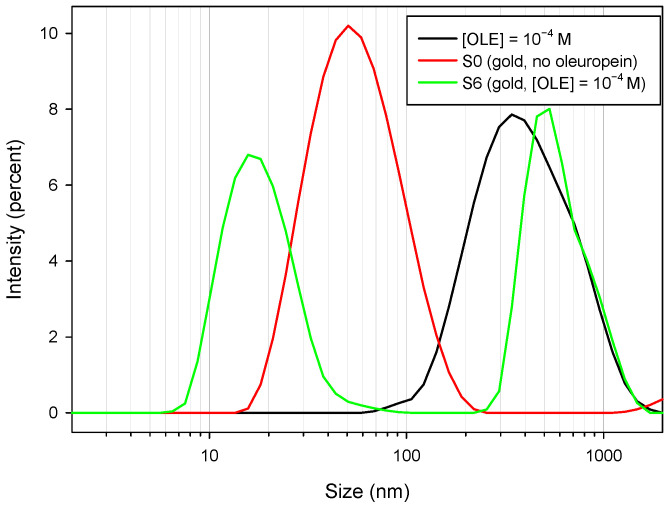
Intensity/size distribution pattern for a solution of [OLE] = 10^−^^4^ M (black line); oleuropein-free borohydride nanoparticles, sample S0 (red line); and sample S6, containing [OLE] = 1 × 10−4 (green line).

In order to ascertain both the existence and the exact nature of the proposed oleuropein self-assembled structures, we performed two additional experiments. As a starting point, DLS measurements were carried out for a small-size sample (S6), a solution of oleuropein in water with the same concentration ([OLE] = 10^−^^4^ M), and a sample containing no oleuropein (S0). Results are shown in Figure 8.

**Figure 9:**
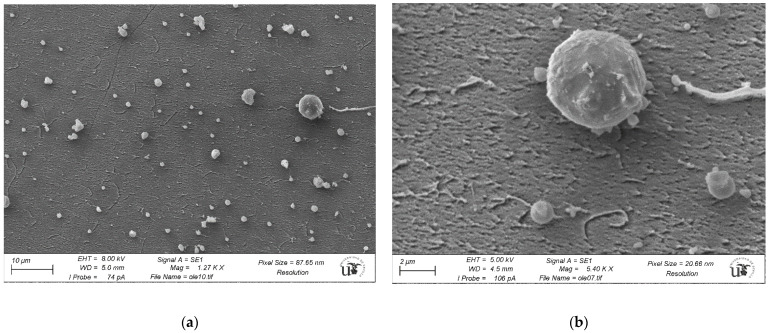
CrioSEM imaging for a solution of [OLE] = 1 × 10^−^^4^ M in water. Left image (**a**) shows a general view, while the right image (**b**) shows the vesicles at a higher magnification. Background noise was due to ice crystal formation.

As a final step, and in order to determine the exact nature of the observed structures, we performed CrioSEM measurements for a solution of [OLE] = 10^−^^4^ M, in the absence of nanoparticles. As evidenced by Figure 9, oleuropein self-assembling occurred even in the absence of any added salts. To our knowledge, this fact has not been reported before, despite oleuropein being a well-known substance. The formed structures were roughly spherical in shape, not dissimilar to vesicles or micelles. As the SEM technique employed had a lower resolution limit of around 200 nm, structures shown tended to be bigger than expected, presenting an average size of 900. Despite this, some smaller bodies of around 300 nm in diameter were also found (see Figure S2). This polydispersity was coherent with the wide peak observed by DLS.

## 4. Discussion

All the nanoparticles synthesized in the present study, except S4 and S0, were found to be stable, without widening, decaying, or shifting of the plasmon band, over at least a 15-day period. S7 to S5 remained stable for a month after synthesis, while S3 to S0 showed slight band-widening and the appearance of red-shifted aggregation bands (approximately 650 nm), indicating that the particles were slowly coagulating over time. S4 showed band-widening and aggregation bands over the initial 15-day period, while S0 quickly decayed after synthesis. The reproducibility of the described synthetic procedures was tested by carrying out two duplicate synthesis containing [OLE] = 1 × 10^−4^ and 5 × 10^−^^5^ M (S6 and S5, see Figure S1). Absorbance spectra of those duplicates, matched out against 48 h spectra of the original sample, showed that both band position and width correlated well between batches, without substantial shifting or widening of the absorbance peaks (see the Supplementary Materials).

S5 and S6 were found to be stable after dialysis, with no band shifts, aggregation bands, or shoulders appearing on the spectra (Figure 4), although a slight red-shifting of the absorbance band was observed, along with a decrease in intensity. Oleuropein absorbance bands, centered around 230-290 nm, greatly diminished upon dialysis, but did not disappear entirely. S0 completely precipitated after 2 h, giving a clear solution as a result. It is interesting to note that dialysis procedures are strongly dependent on the nature of the capping agent—colloids that present covalent bonds and specific interactions that are strong enough will remain unaltered during the procedure, as will those whose capping ligands are big enough not to cross the dialysis membrane, while those who depend upon small ion exchange adsorption equilibriums to remain stable (for example, citrate-capped colloids) will precipitate upon dialyzing. S0 falls into the latter category, as its stabilization is based upon borohydride or borohydride derivatives adsorbing into the gold surface. S5 and S6 belong to the former, a fact that indicates their stabilization is at least partially based upon oleuropein protection. However, as mentioned prior, in-synthesis oleuropein concentrations actually decrease upon dialysis, indicating that the molecule can and does cross the dialysis membrane, but this loss does not result in precipitation of the colloid.

The electrokinetic or zeta potential is directly related to the stability of different kinds of nanoparticles and is linked to counterion concentration and nature in the solution. This electrokinetic potential represents the difference between the compact layer potential and the diffuse potential; due to Coulomb repulsion effects between the colloidal particles, the greater the zeta potential, the more stable the system becomes. In this sense, the magnitude of the measured zeta potential is an indication of the repulsive force that is present and can be used to predict the long-term stability of the nanoparticle. Naturally, it also allows us to obtain information about the proximity of ligands that could neutralize the surface of charged nanoparticles [32,33].

In light of the zeta potential data (Table 2), it becomes clear that the link between surface charge and NaCl aggregation is not immediate—sample S0, which completely precipitated in the presence of NaCl, and samples S5 and S6, which were stable, did present the same negative surface charge potential. As oleuropein was uncharged at neutral pH, the markedly negative values observed should arise from the adsorption of charged borohydride by-products over the particles’ surface; this fact is supported by the observed zeta potential for the borohydride-capped nanoparticles (in the absence of oleuropein) being the same as that observed at the higher oleuropein concentrations. However, borohydride-protected nanoparticles are not dialyzable and completely aggregate in the presence of NaCl, while those obtained in the presence of OLE are perfectly stable both to dialysis procedures and to NaCl. During the dialysis process, a reduction of the concentration of both OLE and borohydride in the synthesis medium took place (see Figure 4). In the case of S0, the lower borohydride concentrations that result caused the system to no longer be stable, while for S6 and S5, the borohydride loss caused no change in zeta potential, and an additional increase of stability to NaCl was found. While all the obtained nanoparticles were ultimately protected by borohydride charges, it was clear that the borohydride–nanoparticle union was substantially strengthened by the presence of the higher concentrations of OLE. As this bond became stronger, dialyzing the system no longer caused it to break, instead increasing stability by reducing ionic strength of the medium. As reported above, this effect was observed to suddenly appear at [OLE] > 5 × 10^−^^5^ M.

The observed increased stability of S7 to S5 to salt aggregation, which became even greater upon dialyzing, points to an enhanced protection of the colloid in the presence of higher oleuropein concentrations. The increased stabilization upon dialysis can be attributed to the ionic strength of the sample decreasing during the procedure. This observed loss of sensitivity to ionic aggregation could have been due to a loss of ionic character of the particles, which would indicate a displacement of negatively charged borohydride molecules from the gold surface by the neutral oleuropein. In this case, the charge-based protection of the colloid would actually be replaced by a steric protective effect.

In addition to being a very useful technique in the nanoscience area, since it allows us to monitor interactions between the nanoparticles and other molecules though changes in the average hydrodynamic diameter, both in static measurements [34,35,36] and in kinetic ones [37], DLS has also found a great niche of application in monitoring aggregation and self-aggregation processes for polymeric and biological samples [38,39,40]. For an example of the application of DLS to synthesis optimization, Zhang et al. determined the most stabilizing aptamer for their colloidal system by considering the decrease of the hydrodynamic size [41,42].

As can be observed, the sample obtained in the absence of oleuropein presented an apparent mean diameter of 50.75 nm through DLS, which was a greater value than that obtained through TEM measurements (35 nm); this difference can be attributed to a number of experimental factors, including laser light absorbance by the measured nanoparticles, and also the influence of the nanoparticles’ outer coating. This effect has been reported for other synthesis procedures employing bulky ligands [43,44]. For the oleuropein solution, a wide peak with a mean size of 342 nm appeared. As oleuropein monomers are too small to correspond to either size, and are in fact not detected by DLS, this peak may correspond to self-assembled oleuropein structures. The sample containing both nanoparticles and oleuropein showed both the aforementioned peaks—although the first was slightly narrower and centered around 531 nm—and a third peak at 15 nm, corresponding to the metal cores. Again, the nanoparticles appeared to have a size greater than the real value.

From DLS measurements, the formation of oleuropein structures seemed to be confirmed. It is interesting to note that, although the nanoparticles showed a negative outer charge (as evidenced by zeta potential measurements; Table 2) due to borohydride adsorption, the formation of the observed structures took place even in the absence of any added ions.

Information obtained through the use of UV–VIS techniques should also be complemented with TEM measurements if aggregation and medium effects, both subtypes of optical phenomena, must be distinguished. TEM measurements give information about the presence of AuNP aggregates and morphological changes of the clusters during the synthesis process in the presence of different ligands [30,45]. Employing air-dried samples ensures the elimination of all possible interferences from the suspension medium. In this case, the most striking feature that can be observed through TEM imaging is the clear shrinking of the mean particle diameter by about 85% upon a fivefold increase on oleuropein concentration, from 10^−^^5^ M (S4) to 5 × 10^−^^5^ M (S5). This change is accompanied by a widening of the absorbance band (Figure 2), a clear darkening of the solution at the naked eye (Figure 1), and increased stability to salt aggregation (Figure 5). Together with the marked nanoparticle morphology changes, all these differences point to a drastic change in oleuropein behavior when its concentration is raised above 10^−^^5^ M. In particular, the smaller size of the particles can be attributed to restricted growth conditions under higher oleuropein concentrations, which in turn points to a possible self-assembly of the oleuropein molecules into supramolecular structures, for example, fibers, membranes, or mono- or multilamellar vesicles. Nanoparticles grown inside those structures would have their growth heavily restricted by the available space, which could help explain the sudden decrease in size. In terms of this, it is important to note that nanoparticle growth in both micelles and multilamellar vesicles has been reported to cause a small population of larger sized particles and anisotropic shapes, mostly triangular and hexagonal [46,47]. Those larger, anisotropic particles with clear straight sides have been found [47] to grow in the free space between structures, which explains both the larger size of the triangular forms and the presence of straight sides, which form on contact points between the growing particle and the structure.

Electron microscopy (scanning, SEM, or transmission, TEM) for analysis of biological samples is a technique that is often used to obtain structural information. SEM has a great advantage in terms of speed, and its interpretation is not complex a priori. However, information is only obtained from the surface of the sample and with a resolution in the range of 1.5 nm. TEM achieves a higher resolution, but the subsequent analysis is much more complex. We must also keep in mind that the need for vacuum in TEM prevents the use of aqueous samples. This implies that frequently, in biological samples, the water is either removed from the sample or replaced by a polymer. In both cases, this almost necessarily implies morphological and structural changes in the sample. The use of CryoSEM has the great advantage of introducing minimal chemical changes, ensuring the samples will always be as close to their original (native) state [48,49,50,51]. Growth inside oleuropein vesicles, such as those observed through CryoSEM and shown in Figure 9, can explain both the small diameter of the nanoparticles obtained in the presence of the higher oleuropein concentrations, and their abnormal stability to both aggregation and dialysis. In contrast to samples S1-S4, obtained at lower oleuropein concentrations and whose protection seemed to mostly be due to borohydride adsorption over the gold surface, samples S5–S7 were protected by an outer coating of oleuropein that lent them added stability at the cost of a smaller size. At a concentration closer to the change limit (5 × 10^−^^5^ M, S5) extravesicular particles with straight faces were also found. As the nanoparticle size was markedly smaller than that of the vesicles that protect them, a double or multiple layered structure could be expected to form, with the particles being trapped inside.

## 5. Conclusions

The stability and structure of a number of novel samples obtained in the presence of oleuropein was analyzed through the use of several techniques. Although all colloids presented a similar anionic character and outer charge, evidencing a borohydride-based protection, definite differences in particle morphology, size, and stability were found for those colloids synthesized at concentrations of oleuropein equal or superior to 5 × 10^−^^5^ M with regards to those obtained in the absence of the compound. Those changes were attributed to the formation of oleuropein self-assembled vesicles, which were observed through the use of CryoSEM imaging.

## Figures and Tables

**Table 1:** Sample nomenclature and oleuropein concentration.

Samples	[OLE]/M
**S0**	0
**S1**	5 × 10^−7^
**S2**	10^−6^
**S3**	5 × 10^−6^
**S4**	10^−5^
**S5**	5 × 10^−5^
**S6**	10^−4^
**S7**	3.3 × 10^−4^

**Table 2:** Mean zeta potential values for all analyzed samples.

Samples	Zeta Potential (mV)
S6	−28.8
S6 (dialyzed)	−29.6
S5	−34.6
S5 (dialyzed)	−29.8
S3	−20.6
S0	−30.7

**Table 3:** Mean particle size of samples S0 and S3 to S7.

Samples	[OLE]/M	Mean Diameter/nm	Polydispersity
**S0**	0	32.8 ± 7.3	22%
**S3**	5 × 10^−6^	35.0 ± 6.6	19%
**S4**	10^−5^	35.0 ± 14.1	40%
**S5**	5 × 10^−5^	5.6 ± 2.0	36%
**S6**	10^−4^	4.6 ± 1.0	22%
**S7**	3.3 × 10^−4^	4.8 ± 0.9	18%

## Data Availability

The data presented in this study are available on request from the corresponding author.

## Supplementary Materials

The following are available online at https://www.mdpi.com/1996-1944/14/1/50/s1: Figure S1: Band comparison between two different batches of synthesis S6 and S5. Figure S2: Size distribution for the oleuropein self-assembled structures observed through CryoSEM.
