# Supplementary material for: Encased Gold Nanoparticle Synthesis as a Probe for Oleuropein Self-Assembled Structure Formation
Source: Materials (Basel). 2020 Dec 24;14(1):50. doi: 10.3390/ma14010050 (PMC7794816; doi:10.3390/ma14010050)
Supplement: Supplementary file 1 [file materials-14-00050-s001.zip › materials-1008436-supplementary.pdf]

materials

Electronic Supplementary Information (ESI)

Encased Gold Nanoparticle Synthesis as a Probe for Oleuropein Self-Assembled Structure Formation

Aila Jimenez-Ruiz *[,1], Rafael Prado-Gotor *[,1], José G. Fernández-Bolaños [2], Alejandro González-Benjumea [2] and Jose M. Carnerero [1]

[1] Department of Physical Chemistry, University of Seville. c/Profesor García González 1, 41012 Seville, Spain; jcarnerero2@us.es
[2] Department of Organic Chemistry, University of Seville. c/Profesor García González 1, 41012 Seville, Spain; bolanos@us.es (J.G.F.-B.); agonzalez15@us.es (A.G.-B.)
* Correspondence: ailjimrui@alum.us.es (A.J.-R.); pradogotor@us.es (R.P.-G.)

Synthesis reproducibility data

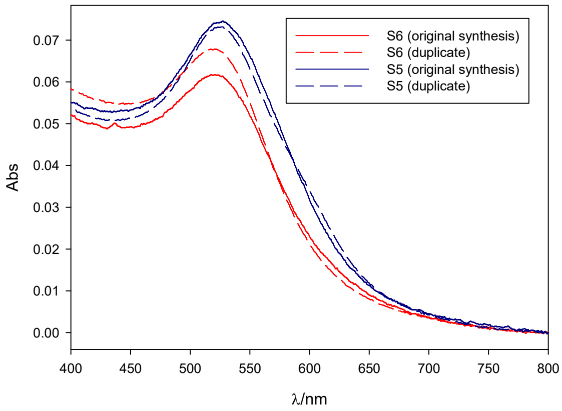

Figure S1. Band comparison between two different batches of synthesis S6 and S5.

CryoSEM - Oleuropein vesicle size measurements

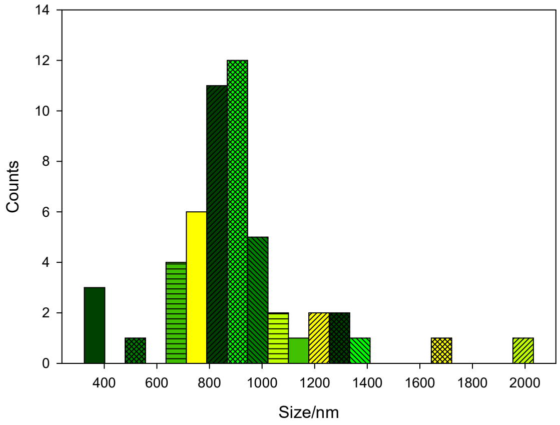

Figure S2. Size distribution for the oleuropein self-assembled structures observed through CryoSEM.
